# Can aspartate aminotransferase and platelet distribution width-to-platelet ratio in the first trimester predict fetal macrosomia?: a retrospective case-control study

**DOI:** 10.1590/1806-9282.20240919

**Published:** 2025-03-17

**Authors:** Fahri Burcin Firatligil, Arife Akay, Merve Ugur, Sadun Sucu, Yıldız Akdas Reis, Serap Topkara Sucu, Yaprak Engin-Ustun

**Affiliations:** 1 Ankara Etlik City Hospital, Department of Obstetrics and Gynecology, Division of Perinatology – Ankara, Turkey.; 2 Ankara Etlik Zubeyde Hanim Women's Health Education and Research Hospital, Department of Obstetrics and Gynecology – Ankara, Turkey.; 3 Ankara Etlik City Hospital, Department of Obstetrics and Gynecology – Ankara, Turkey.

**Keywords:** AST, Fetal macrosomia, First pregnancy trimester, Biomarkers, Platelet

## Abstract

**OBJECTIVE:**

The aim of this study was to investigate the performance of aspartate aminotransferase level and platelet distribution width-to-platelet ratio as predictive factors for fetal macrosomia in the first trimester.

**METHODS:**

This retrospective case-control study was conducted between August 2017 and August 2020. The data of the study group as Group I (n=426) and the control group as Group II (n=426) were collected and compared by scanning the records. For each patient who was eligible for Group I, the first patient from the file review who met the criteria listed in the inclusion/exclusion section was selected for Group II. Aspartate aminotransferase levels and serum platelet distribution width-to-platelet ratio levels were determined in the first trimester using the participants’ medical records. The study parameters of the two groups were statistically compared.

**RESULTS:**

The median aspartate aminotransferase, platelet, platelet distribution width, and platelet distribution width-to-platelet ratio values of the laboratory test results in the first trimester were significantly different. The aspartate aminotransferase and platelet distribution width-to-platelet ratio values were higher in Group I.

**CONCLUSION:**

Higher aspartate aminotransferase and platelet distribution width-to-platelet ratio levels in the maternal blood sample in the first trimester indicate an unbalanced inflammatory process causing fetal macrosomia. The cutoff values for aspartate aminotransferase (>21 U/L) at 94% specificity and for platelet distribution width-to-platelet ratio (>0.19) at 51% specificity can be used as markers for a screening test. However, randomized controlled trials combining body mass index and the parameters in the present study are needed in future studies.

## INTRODUCTION

Fetal macrosomia (FM) is a terminology used to describe a newborn with an extreme birth weight (BW)^1^. This term has been defined in various ways, including as a BW greater than 4000 g or greater than 90% for gestational age^1,2^. A 2017 report on births in the United States concluded that approximately 7.8% of infants had a BW greater than 4000 g, 1% had a BW greater than 4500 g, and 0.1% had a BW greater than 5000 g^3^.

Risk factors for FM are associated with gestational diabetes mellitus (GDM), high body mass index (BMI), extreme weight gain during pregnancy, types of diabetes mellitus (DM), genetic factors, and so on^1^. FM is usually considered a high-risk pregnancy and requires intensive antenatal care^1^, which can lead to maternal, fetal, and neonatal complications^1^. Maternal risks are associated with a higher incidence of cesarean section (CS), perineal tears, postpartum hemorrhage, labor protraction, and labor arrest^1,4,5^; fetal and neonatal risks are also associated with stillbirth, shoulder dystocia, and birth trauma due to FM^5^.

Given the higher rates of these adverse pregnancy outcomes, the prediction of FM in pregnancy is particularly important. Our hospital is a tertiary referral center with more than 20,000 pregnancies per year coming for first-trimester screening tests. Therefore, we have a good chance and a good population to obtain useful data. Thyroid and kidney function tests and liver enzyme tests are routinely performed on pregnant women admitted to the perinatology department in the first trimester. For the prediction of FM, we estimate the performance of aspartate aminotransferase (AST) level and platelet distribution width (PDW)-to-platelet (PLT) ratio (PDW/PLT) in the first trimester as predictive factors for FM.

## METHODS

This retrospective case-control trial was conducted between August 2017 and August 2020 in the Perinatology Department of Etlik Zubeyde Hanim Women's Health Education and Training Hospital.

### Inclusion and exclusion criteria

Screening for FM was based on the term defined according to the standards of the American College of Obstetricians and Gynecologists^1^. This study categorized uncomplicated, singleton, nulliparous pregnant women aged 18–40 years with FM (confirmed postnatal macrosomia) as the study group and uncomplicated, singleton, nulliparous pregnant women aged 18–40 years with non-FM (confirmed postnatal non-macrosomia) as the control group.

Women with post-term pregnancies; pregnant women admitted to the hospital for preterm labor and preterm premature rupture of membranes; pregnant women with GDM or DM Types I and II; pregnant women admitted to the hospital for reasons such as fetal growth restriction (FGR); women with hypertensive pregnancy disorders; pregnant women with chronic diseases; pregnant women with liver dysfunction; women with multiple pregnancies; pregnant women aged <18 and >40 years; and pregnant women with a BMI<25 kg/m^2^ and >40 kg/m^2^ (to standardize the maternal BMI between normal, healthy, and morbidly obese weight) were excluded from the study.

### Data

The power analysis was performed with the program G*Power 3.1^6^. A sample of approximately 426 cases (pregnant women delivering a macrosomic fetus) and 426 controls (pregnant women delivering a non-macrosomic fetus) was required with a power of 80% and an α-level of 0.05^7^. Thus, the present study included a total of 852 pregnant women.

Data were extracted from the patient records or hospital records of both groups, including participants’ demographic information; anthropometric parameters; educational status; smoking status; AST, PDW, and PLT values in the first trimester; fetal sex; fetal BW; and mode of delivery. In our hospital, height and weight are measured in a standardized manner at the patient's first visit to the nurse's office in the first trimester and recorded in the patient's medical record to calculate BMI. These data were taken from the patients’ records and were included in the study.

### Study design

’The data of the Group I (those who delivered a macrosomic fetus) (n=426) and the Group II (those who delivered a non-macrosomic fetus) (n=426) were retrospectively collected and compared by scanning the records.. For each patient who was eligible for Group I, the first patient from the file review who met the criteria listed in the inclusion/exclusion section was selected for Group II.

AST and serum PDW/PLT levels were determined in the first trimester using the participants’ medical records. The study parameters of the two groups were statistically compared.

### Laboratory analysis of biological samples

AST, PDW, and PLT levels obtained from the participants’ medical records were analyzed using the Advia 2400 clinical chemistry system (Siemens, Tarrytown, NY, USA).

### Statistical analysis

Statistical analyses of the results were performed using IBM SPSS Statistics program, version 24.0^8^. The Levene test was used to assess the homogeneity of variance. Descriptive analyses were presented using means and standard deviations for variables that fit the normal distribution. The t-test for independent samples was used to compare these parameters between the groups. Means and quartiles (Q1-Q3) were used for numerical data that did not conform to the normal distribution. Mann-Whitney U tests were used to compare these parameters between the groups. Descriptive analyses for categorical variables were presented using frequencies and percentages. Associations between categorical variables were analyzed using the chi-square test or Fisher's exact test (if the assumptions of the chi-square test were not valid due to low expected cell counts). The ability of AST and PDW/PLT to predict FM was analyzed using receiver operating characteristic (ROC) curve analysis. Sensitivity, specificity, and area under the curve (AUC) were reported when a significant cutoff value was found. A p<0.05 was considered statistically significant.

## RESULTS

**Figure 1:**
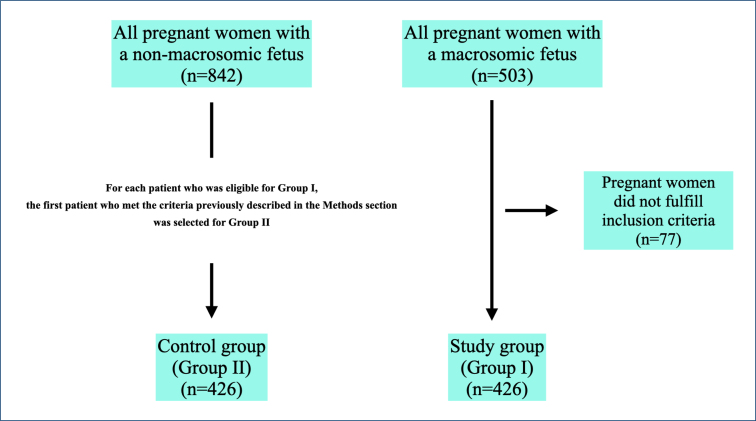
The flowchart of the participants.

On analyzing patient and/or hospital records, 503 pregnant women with macrocosmic fetus were identified (Figure 1). After screening according to the criteria described in the "Methods" section, 426 pregnant women with FM were included in Group I. For each patient who was eligible for Group I, the first patient who met the criteria previously described in the "Methods" section was selected for Group II.

A comparison of the demographic data and laboratory test results between the groups is shown in Table 1. There were no significant differences between the groups with respect to maternal age, BMI, smoking status, employment status, gravidity, parity, abortion, and week of gestation at the time of blood sampling. Educational status, mode of delivery, fetal BW values, and fetal sex were statistically significant (p<0.001). The median AST, PLT, PDW, and PDW/PLT values were significantly different for laboratory test results in the first trimester (p<0.001, p<0.001, p=0.004, and p<0.001, respectively).

**Table 1:** Comparison of demographic data and laboratory test results between the groups

Variables	Group I (n=426)	Group II (n=426)	p-value	
Age (years)	28 (24-32)	27 (24-32)	0.432	
BMI (kg/m^2^)	28 (26-30)	28 (25-31)	0.840	
Smoking status (yes)	79 (18.5)	69 (16.4)	0.366	
Working status (yes)	129 (30.3)	118 (27.7)	0.406	
Educational status				
Primary	7 (1.6)	51 (12)	**<0.001**	**<0.001**
Secondary	90 (21.1)	58 (13.6)	**0.004**	
High	235 (55.2)	170 (39.9)	**<0.001**	
University	94 (22.1)	147 (34.5)	**<0.001**	
Gravidity	2 (1-4)	2 (1-4)	0.140	
Parity	1 (0-2)	1 (0-2)	0.063	
Abortion	0 (0-1)	0 (0-1)	0.940	
WGBS (weeks)	8 (7-10)	8 (7-10)	0.844	
Mode of delivery				
SVD	143 (33.6)	227 (53.3)	**<0.001**	
CS	283 (66.4)	199 (46.7)		
Fetal sex				
Male	303 (71.1)	239 (56.1)	**<0.001**	
Female	123 (28.9)	187 (43.9)		
BW (g)	4190 (4090-4330)	3640 (3340-3680)	**<0.001**	
AST (U/L)	17 (15-22)	15 (13-18)	**<0.001**	
PLT (×10^9^/L)	240 (192-270)	252 (220-295)	**<0.001**	
PDW (fL)	52 (47-56)	51 (45-56)	**0.004**	
PDW/PLT	0.221 (0.184-0.286)	0.194 (0.154-0.243)	**<0.001**	

AST: aspartate aminotransferase; BMI: body mass index; BW: birth weight; PDW: platelet distribution width; PLT: platelet; WGBS: week of gestation in which the blood sample was taken; PDW/PLT: platelet distribution width-to-platelet ratio; CS: cesarean section; SVD: spontaneous vaginal delivery. Statistically significant values are denoted in bold.

The ROC curve analysis to evaluate the performance of AST and PDW/PLT values in predicting FM is shown in Table 2. According to the ROC curves plotted using Youden index results, the optimal sensitivity/specificity value was >21 U/L for AST (27% sensitivity and 94% specificity) and >0.19 for PDW/PLT (68% sensitivity and 51% specificity).

**Table 2:** The ROC curve analysis to evaluate the performance of AST and PDW/PLT values in predicting fetal macrosomia

Variables	AUC	95%CI	Cutoff	p-value	Sensitivity	Specificity
BMI	0.504	0.470-0.538	>30	0.842	17	73
AST	0.659	0.626-0.691	>21	**<0.001**	27	94
PLT	0.611	0.577-0.644	≤202	**<0.001**	32	84
PDW	0.557	0.523-0.591	>52	**0.004**	48	64
PDW/PLT	0.634	0.600-0.666	>0.19	**<0.001**	68	51

AST: aspartate aminotransferase; AUC: area under curve; BMI: body mass index; CI: confidence interval; PDW: platelet distribution width; PLT: platelet; PDW/PLT: platelet distribution width-to-platelet ratio. Statistically significant values are denoted in bold.

## DISCUSSION

To the best of our knowledge, the physiopathology of FM depends on the associated maternal or fetal conditions. In general, maternal obesity, extreme maternal weight gain during pregnancy, and uncontrolled DM are often associated with fetal weight gain, leading to FM^9^. Hyperglycemia in the fetus leads to the activation of insulin and other growth factors that stimulate fetal growth and accumulation of fat and glycogen^10^. In addition, advanced gestational age allows the growth process to continue in utero, resulting in higher fetal weight at birth^9^.

Various studies have investigated the in utero development of FM and tried to get some preliminary information about FM prediction. Poon et al.^11^ reported that the combinations of maternal characteristics (BMI, smoking status, chronic diseases, ethnic origin, etc.) and first-trimester screening test (pregnancy-associated plasma protein A, free b-human chorionic gonadotropin, and nuchal translucency measurement) parameters can be used in FM prediction. Zbucka-Kretowska et al.^12^ investigated the diagnostic value of first-trimester adipokines and placental markers in predicting FM. The authors concluded that irisin, an adipokine, could be a biomarker for the early prediction of FM^12^. Another study conducted for the prediction of FM in the first trimester concluded that parameters such as (a) BMI above 25.5 kg/m^2^; (b) adiponectin and soluble E-selectin serum concentrations; and (c) maternal weight cutoff at the time of first-trimester screening test of 67 kg had a high detection capacity for FM^13^.

AST is an enzyme whose high serum levels may indicate liver damage^14^; there are also studies showing that high AST levels are associated with an increased risk of Type II DM. It is well known that Type II DM is one of the causes of FM^14^. In English-language medical research, PDW, PLT, and PDW/PLT are used as indices for the clinical and pathophysiologic assessment of vascular diseases (including preeclampsia) and the severity of diseases (inflammation), but their value is not yet fully known^15^. Some studies have shown that PDW/PLT levels decrease with inflammation^16,17^. In obstetrics, several studies have shown that inflammation and vascular diseases are particularly associated with FGR^18,19^.

Consequently, pregnancy-related inflammatory responses fulfill a dual purpose: they support the healthy course of pregnancy and contribute to pathology in the case of dysregulation^20^. Controlled inflammation is essential for the formation of the placenta and implantation of the embryo in early pregnancy^20^. During pregnancy, a regulatory environment is created to protect the fetus from the excessive response of the maternal immune system^20^. However, an imbalance in anti-inflammatory factors can lead to complications, such as FGR, FM, preterm delivery, and GDM^20^. The results of our study are similar to the findings in the review by Ray et al.^20^ and suggest that unbalanced inflammation may cause FM.

In conclusion, higher AST and PDW/PLT levels in the maternal blood sample in the first trimester indicate an unbalanced inflammatory process causing FM. The cutoff values for AST (>21 U/L) at 94% specificity and for PDW/PLT (>0.19) at 51% specificity can be used as markers for screening tests. With an AST value of >21 U/L or a PDW/PLT level of >0.19, physicians should be more cautious about the risk of FM. Although the specificity of PDW/PLT at a value of >0.19 is 51% in pregnant women at risk of FM, the study by Huda et al.^21^ on obesity and FM showed a relative risk of 2.36, which is more significant than the PDW/PLT ratio in our study. Therefore, randomized controlled trials combining BMI and the parameters in the present study are needed in future studies.

### Strengths and limitations

Standardized protocols were used for all pregnant women in this study, and the groups were homogeneously distributed. Furthermore, AST and PDW/PLT were used for the first time in the literature to predict FM and are more cost-effective tests than other costly proteins such as irisin and E-selectin, which were previously used to predict FM. All pregnant women were cared for and delivered in the same referral hospital.

However, at a level of >0.19 of PDW/PLT, the specificity with a PDW/PLT value of >0.19 is 51%, but the limitations of this value are the experience of a single center and the retrospective study design..

### Recommendations to physicians and health policies

AST and complete blood count in the first trimester allow physicians to determine the risk of FM during this period in addition to fasting blood glucose.. The recommendations for screening for GDM in early pregnancy vary widely^22^. Hence, pregnant women who are at risk of FM can start changing their lifestyle and diet immediately. In addition, universal ultrasound screening should be performed in the third trimester to detect pregnancies with FM. Pregnant women should be informed of the adverse maternal and fetal complications of FM, although it is known that about half of the complications, such as shoulder dystocia, occur with a BW below 4000 g. Pregnant women with FM may be offered CS as an option for the mode of delivery.

A serious fetal complication, such as permanent brachial plexus injury, will lead to many lawsuits against obstetricians aimed at lowering the primary CS rate. Critical litigation may also arise for clinicians who rely on a threshold to terminate most births with CS to avoid these complications. Therefore, a central threshold and guideline should be officially announced by the national Ministry of Health to prevent an increase in the cost of obstetric care and protect trusted obstetricians. On the other hand, patients and/or their partners who do not accept the reasonable increased risk of mild and transient neonatal complications should have the right to decide on the mode of delivery in such critical situations at the expense of their own health insurance.
